# Phosphate Ceramics with Silver Nanoparticles for Electromagnetic Shielding Applications

**DOI:** 10.3390/ma15207100

**Published:** 2022-10-13

**Authors:** Edita Palaimiene, Jan Macutkevič, Jūras Banys, Algirdas Selskis, Natalia Apanasevich, Alexander Kudlash, Aliaksei Sokal, Konstantin Lapko

**Affiliations:** 1 Physics Faculty, Vilnius University, Sauletekio Av. 9, LT-10222 Vilnius, Lithuania; 2 Center for Physical Science and Technology, Sauletekio Av. 3, LT-10257 Vilnius, Lithuania; 3 Independent Researchers, 220002 Minsk, Belarus

**Keywords:** microwave shielding, silver nanoparticles, phosphate ceramics, electrical transport

## Abstract

Ceramic composites with nanoparticles are intensively investigated due to their unique thermal, mechanic and electromagnetic properties. In this work, dielectric properties of phosphate ceramics with round silver nanoparticles of various sizes were studied in the wide frequency range of 20 Hz–40 GHz for microwave shielding applications. The percolation threshold in ceramics is close to 30 wt.% of Ag nanoparticles content and it is higher for bigger-sized nanoparticles. The microwave complex dielectric permittivity of ceramics above the percolation threshold is rather high (ε′ = 10 and ε″ = 10 at 30 GHz for ceramics with 50 wt.% inclusions of 30–50 nm size, it corresponds to almost 61% absorption of 2 mm-thickness plate) therefore these ceramics are suitable for microwave shielding applications. Moreover, the microwave absorption is bigger for ceramics with a larger concentration of fillers. In addition, it was demonstrated that the electrical transport in ceramics is thermally activated above room temperature and the potential barrier is almost independent of the concentration of nanoparticles. At very low temperature, the electrical transport in ceramics can be related to electron tunneling.

## 1. Introduction

Nowadays, electromagnetic radiation is widely used in all areas of human life, including telecommunications and electronics [1]. Electromagnetic interference becomes a very important problem because some devices work in the same or neighborhood frequency ranges; in addition, the power of electromagnetic radiation increases with the number of pieces of equipment functioning in a close space and by fast progress of telecommunications, where higher frequencies and hence higher power are needed for transmission large quantity of information. Moreover, electromagnetic interference can cause the destruction of electronic devices and can be dangerous to human health. 

Conductive metal nanomaterials are very important for fabrication of composites and coatings, since these particles have a big surface area and outstanding mechanical and optical properties [2]. Ceramics with conductive nanoparticles have thermally stable electromagnetic properties and excellent mechanical properties [3,4]. Therefore they are highly desirable for various electromagnetic shielding applications [5,6]. One most important parameter, which determines the electromagnetic properties of the composite insulator–conductor system, is the percolation threshold, the lowest concentration of fillers when the electrical conductivity is observed [7]. It is desirable that the percolation threshold will be as low as possible. The percolation threshold is strongly dependent on the particle aspect ratio and the distribution of nanoparticles in the matrix. Theoretically, the lowest percolation threshold is expected for prolonged nanoparticles, i.e., for carbon nanotubes [8]. Moreover, experimentally low percolation threshold values are observed in composites with various carbon nanoinclusions: carbon nanotubes, carbon black, onion-like carbons and graphene [8,9,10,11]. These composites are widely investigated for electromagnetic shielding applications [12,13,14]. Moreover, the percolation threshold can be quite low for composites with round metallic nanoparticles [15]. Particularly, the percolation threshold can be quite low in composites with silver nanoparticles, therefore composites with these nanoparticles can be interesting for electromagnetic applications [16,17]. Moreover, composites with silver nanoparticles are widely used for electromagnetic shielding applications [18,19]. However, electromagnetic properties of ceramics with silver nanoparticles have not been investigated up to now. The aim of this paper is to investigate broadband properties of ceramic composites with silver nanoinclusions for electromagnetic shielding applications.

## 2. Materials and Methods

Phosphate ceramic composites with two kind of commercially available spherical silver nanoparticles (30–50 nm and 80–100 nm) [20,21] were fabricated as follows. Phosphate composite materials consisted of three components: main filler, functional additive (i.e., functional filler) and binder. At first step, the main filler was obtained by thorough mixing of powdered aluminum oxide Al_2_O_3_ and aluminum nitride AlN with a mass ratio equal to 9:1. Then, the main filler, Ag nanopowders, solid magnesium phosphate binder (Mg(H_2_PO_4_)_2_·2H_2_O, designated as MPB) and distilled water were thoroughly mixed for 25–30 min at room temperature (21 ± 1 °C) until a homogeneous composition was obtained. It was experimentally found that the optimal amounts of MPB and water were 20 and 10 wt.%, respectively. The content of Ag nanoparticles in the phosphate composites was varied and fixed at 10, 20, 30, 40 and 50 wt.%. After mixing all the components, the phosphate composites were pressed at 4.9 MPa into tablets 12 mm in diameter and 1–2 mm of thickness (for carrying out further conductivity and electromagnetic measurements). As-fabricated composite tablets were kept for 24 h at ambient room air temperature and then thermal treatment was performed at up to 200 °C with a heating rate of 1 °C/min. 

Scanning electron microscopy (SEM) was performed on a Helios Nanolab 650 microscope. The dielectric properties were investigated with an LCR meter, the HP4284A, in the frequency range 20 Hz–1 MHz. The equivalent circuits for investigations were selected as the capacitance and the loss tangent circuits. The complex dielectric permittivity was calculated according to the planar capacitor equations [22]. For the low temperature measurements, a cryostat with liquid nitrogen was used; while for higher temperatures a home-made furnace was used. Each measurement was started at room temperature. Silver paste was applied for electric contact. In microwave frequency range (26–35 GHz), the dielectric properties were determined by a thin cylindrical rod method, measuring reflection and transmission modulus [22]. In this frequency range measurements were performed with a scalar network analyzer, the R2-408R.

## 3. Results

### 3.1. Structure of Ceramics and Room Temperature Properties

**Figure 1:**
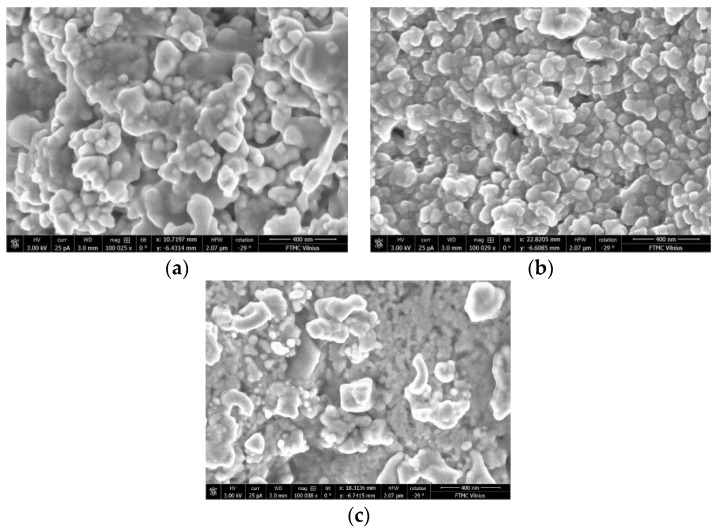
SEM images of phosphate ceramics samples with Ag inclusions of different size and concentrations: (**a**) 50 wt.% of 30–50 nm nanoparticles, (**b**) 30 wt.% of 80–100 nm nanoparticles, (**c**) 50 wt.% of 80–100 nm nanoparticles.

SEM images of phosphate ceramics with different Ag particle sizes and concentrations are presented in Figure 1. The study of the surface morphology of prepared composite samples showed that Ag particles were quite homogeneously distributed in the ceramics matrix, independent of their size and concentration.

**Figure 2:**
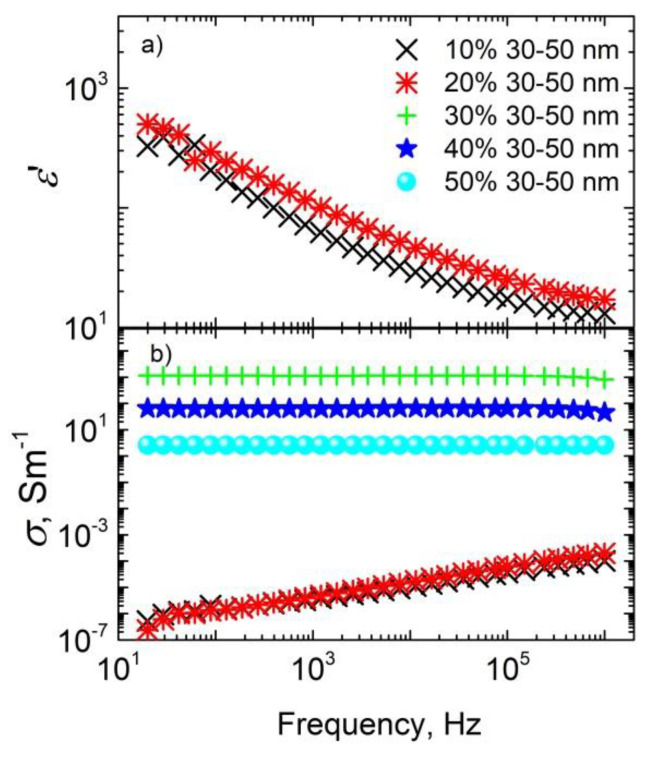
Room-temperature dielectric permittivity (**a**) and electrical conductivity (**b**) of phosphate ceramics with Ag inclusions (30–50 nm nanoparticles) plotted as a function of frequency.

**Figure 3:**
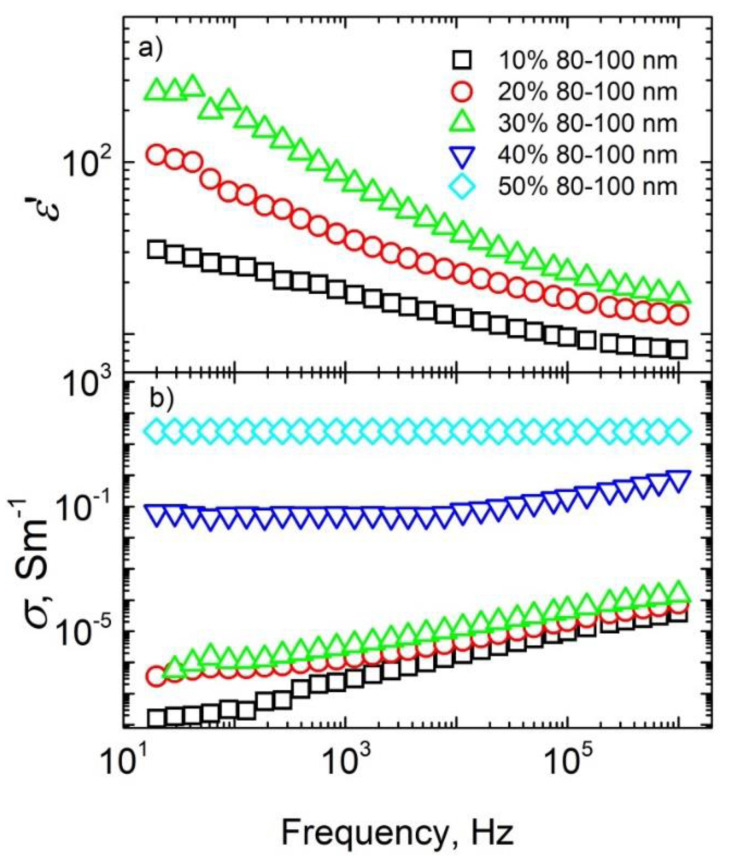
Room-temperature dielectric permittivity (**a**) and electrical conductivity (**b**) of phosphate ceramics with Ag inclusions (80–100 nm nanoparticles) plotted as a function of frequency.

The frequency dependences of both dielectric permittivity and electrical conductivity for different Ag aggregate sizes at room temperature in the frequency range 20 Hz–1 MHz are presented in Figure 2 and Figure 3. 

For some ceramics, the electrical conductivity is frequency-independent at low frequencies; this effect was particularly observed for ceramics with 30–50 nm sized nanoparticles and concentrations not lower than 30 wt.%, and for ceramics with 80–100 nm sized nanoparticles and concentrations not lower than 40 wt.%. The frequency-independent conductivity at low frequencies is related to DC conductivity. Therefore, the above-indicated concentrations (30 wt.% for ceramics with 30–50 nm sized nanoparticles and 40 wt.% for ceramics with 80–100 nm sized nanoparticles) were close to the percolation threshold in the systems. Thus, the percolation threshold was higher for ceramics with bigger nanoparticles. Such a percolation threshold value is in agreement with excluded volume theory [23,24], while the increasing of the percolation threshold with nanoparticles size can be explained by the better distribution of nanoparticles inside ceramic matrices. Unfortunately, the dielectric permittivity in the frequency range 20 Hz–1 MHz was determined only below the percolation threshold, because at higher Ag nanoparticle concentrations tgδ is very high and the dielectric permittivity cannot be determined. Below the percolation threshold, the dielectric permittivity values were quite low (about 10–20 at 1 MHz) and were almost concentration-independent. Electrical conductivity values under such conditions were also quite low and were strongly dependent on frequency.

**Figure 4:**
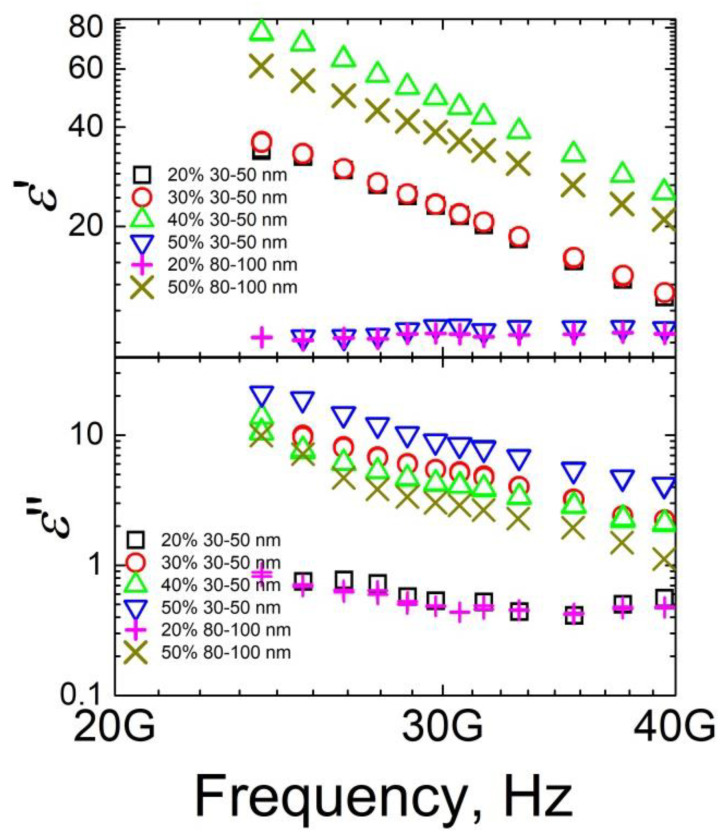
Frequency spectra of the complex dielectric permittivity real and imaginary parts for phosphate ceramics with Ag nanoinclusions in microwave frequency range at room temperature.

Frequency dependences of real and imaginary parts of dielectric permittivity for the investigated ceramics in microwave frequency range are presented in Figure 4. Above the percolation threshold, values of real and imaginary parts of dielectric permittivity were quite high (ε′ = 46 and ε″ = 8 at 30 GHz for ceramics with 40 wt.% inclusions of 30–50 nm size), therefore ceramics above the percolation threshold can be suitable for electromagnetic shielding applications [3,4,5]. Indeed, if we consider a thin planar layer in free space with the electromagnetic irradiation incident perpendicularly, then the scattering parameters can be calculated according to Equations (1) and (2):

S_11_ = −j[(k_z_/k_2z_)^2^ − 1]sin(k_2z_τ)/(2jk_z_/k_2z_cos(k_2z_τ) + [(k_z_/k_2z_)^2^ + 1]sin(k_2z_τ), (1)

S_21_ = 2(k_2z_/k_z_)/(−2(k_2z_/k_z_)cos(k_2z_τ) + j[(k_2z_/k_z_)^2^ + 1]sin(k_2z_τ)), (2)

where k_z_ = 2π/λ and k_2z_ = 2πε^0.5^/λ are wave numbers in the vacuum and the sample’s media correspondingly, and τ is the thickness of the layer. The absorption of the layer was calculated as A = 1 − (S_11_)^2^ − (S_21_)^2^. Obtained calculations results for ceramics layers with thickness 2 mm at 30 GHz are summarized in Table 1. It can be concluded that the absorption of some ceramics (for example, the absorption of ceramics with 50 wt.% inclusions of 30–50 nm size is 61%) was quite high. Moreover, by application of the Salisbury screen method [25] to the presented ceramics it was possible to obtain even 100% absorption. These results are better than those previously reported for ceramics with SiC nanoinclusions or carbon nanotubes [3,4,26]. Although values of complex dielectric permittivity decrease with frequency according to the Jonscher universal power law [27,28], they increase with nanofiller concentration. Thus, microwave transmission is lower for ceramics with higher filler concentration.

### 3.2. Electrical Transport at Different Temperatures

**Figure 5:**
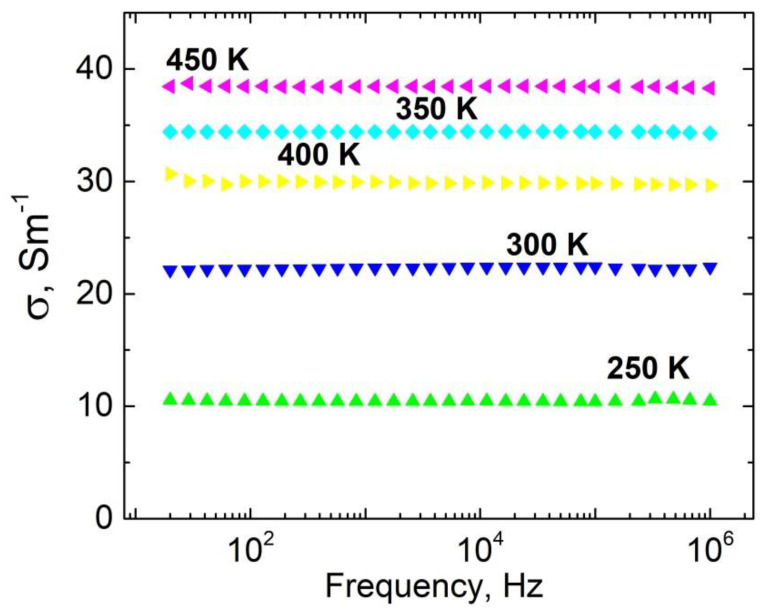
Frequency spectra of electrical conductivity for phosphate ceramics with 50 wt.% Ag nanoinclusions (size 80–100 nm).

Frequency spectra of electrical conductivity for ceramics with 50 wt.% Ag nanoinclusions (80–100 nm) are presented in Figure 5. From these spectra is possible to conclude that the AC conductivity coincided with DC conductivity values in the frequency range 20 Hz–1 MHz. Additionally, the electrical conductivity changed several times in the temperature range 250–450 K, which is important for evaluation of the thermal stability of coatings for electromagnetic shielding applications.

**Figure 6:**
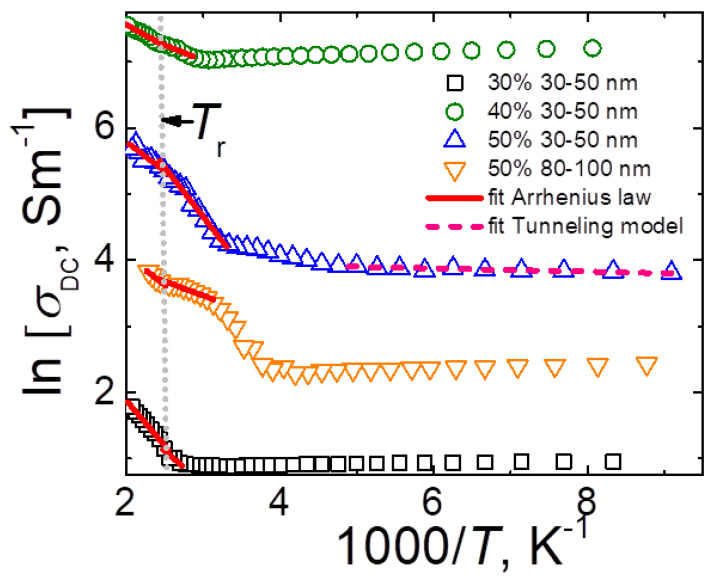
Inverse temperature dependence of DC conductivity for phosphate ceramics with Ag nanoinclusions.

The temperature dependence of DC conductivity is presented in Figure 6. Three different regions can be separated: (1) in 300–500 K temperature range the electrical conductivity increased with temperature due to the thermal activation mechanism [29], (2) below room temperature the electrical conductivity increased on cooling due to rapid shrinkage of ceramics, (3) and at very low temperatures (below 200 K) for ceramics with 50 wt.% Ag nanoparticles of 30–50 nm the electrical conductivity decreased on cooling due to the electron tunneling effects [30]. Above room temperature, the temperature dependence of DC conductivity fitted with the Arrhenius law:

(3) $$\sigma =\sigma_{0}e^{-\frac{E}{\mathrm{kT}}}$$

where σ_0_ is the pre-exponential factor, k is the Boltzmann constant and E is the activation energy. Because ln{σ_DC_}(1/T) has a slope close to temperature T_r_, it was fitted separately below and above this temperature. Obtained parameters are summarized in Table 2. The activation energy value was about several tenths of meV and it was almost independent of filler concentration. This is typical for thermally activated electrical conductivity [29].

The DC conductivity data for temperatures below 220 K fitted well to the fluctuation-induced tunneling (FIT) model [30]:

(4) $$\sigma =\sigma_{0}e^{-\frac{E}{k\left(T+T_{0}\right)}}$$

T_1_ = ωAε^2^_0_/8πk, (5)

T_0_ = Aε^2^_0_/4π^2^χk, (6)

where ω is the width of the tunneling gap, A is the area of the capacitance formed by the tunnel junction, ε_0_ = 4V_0_/ew, where V_0_ is the potential barrier height, e is the electron charge, and χ = (2mV_0_/h^2^)^1/2^, where m is the electron mass and h is Planck’s constant. The tunneling law fit parameters for ceramics with 50 wt.% of silver nanoparticles of 30–50 nm size were σ_0_ = 56.68 mS/m, T_1_ = 28.4 K, T_0_ = 11.7 K, T_1_/T_0_ = 2.43. We assume that for other ceramics under investigation, the tunneling conductivity also should be observed; however, at substantially lower temperatures (below the lowest possible temperature in our measurements) due to the bigger potential barrier height.

## 4. Conclusions

Broadband dielectric investigations of phosphate ceramics with round silver nanoparticles of various sizes indicated that the percolation threshold was close to 30 wt.% of Ag nanoparticles, which is typical for composites with round nanoparticles, and it was bigger for bigger nanoparticles. The microwave complex dielectric permittivity of ceramics above the percolation threshold was rather high (ε′ = 10 and ε″ = 10 at 30 GHz for ceramics with 50 wt.% inclusions of 30–50 nm size: it corresponded to 61% absorption of 2 mm thickness plate). Therefore, these ceramics are suitable for microwave shielding applications, for example, in Salisbury screen geometry, where thermally stable coatings with good mechanical properties are needed. Moreover, it was found that the electrical transport in ceramics is thermally activated above room temperature and the potential barrier was almost independent of the concentration of nanoparticles. At very low temperature, the electrical transport in ceramics can be related to electron tunneling.

## Figures and Tables

**Table 1:** Reflection, transmission and absorption of phosphate ceramics with Ag nanoinclusions of 2 mm thickness calculated at 30 GHz.

Concentration and Average Size of Ag Nanoparticles	Reflection	Transmission	Absorption
20% 30–50 nm	0.39	0.45	0.16
30% 30–50 nm	0.39	0.08	0.53
40% 30–50 nm	0.59	0.07	0.35
50% 30–50 nm	0.38	0.01	0.61
20% 80–100 nm	0.13	0.62	0.25
50% 80–100 nm	0.75	0.08	0.17

**Table 2:** Fit parameters of the temperature dependence of DC conductivity in different temperatures ranges.

Ag (wt.%), Size	Temperature Region	E, eV	ln(σ_0_, Sm^−1^)
30% 30–50 nm	T > T_r_	0.118	4.62
	T < T_r_	0.086	3.62
40% 30–50 nm	T > T_r_	0.056	8.86
	T < T_r_	0.041	8.43
50% 30–50 nm	T > T_r_	0.077	7.57
	T < T_r_	0.130	9.17
50% 80–100 nm	T > T_r_	0.079	5.91
	T < T_r_	0.037	4.76

## Data Availability

Not applicable.
